# Treatment of Primary Aldosteronism and Reversal of Renin Suppression Improves Left Ventricular Systolic Function

**DOI:** 10.3389/fendo.2022.916744

**Published:** 2022-06-30

**Authors:** Troy H. Puar, Chin Kai Cheong, Roger S.Y. Foo, Seyed Ehsan Saffari, Tian Ming Tu, Min Ru Chee, Meifen Zhang, Keng Sin Ng, Kang Min Wong, Andrew Wong, Foo Cheong Ng, Tar Choon Aw, Joan Khoo, Linsey Gani, Thomas King, Wann Jia Loh, Shui Boon Soh, Vanessa Au, Tunn Lin Tay, Eberta Tan, Lily Mae, Jielin Yew, Yen Kheng Tan, Khim Leng Tong, Sheldon Lee, Siang Chew Chai

**Affiliations:** ^1^ Department of Endocrinology, Changi General Hospital, Singapore, Singapore; ^2^ Yong Loo Lin School of Medicine, National University of Singapore, Singapore, Singapore; ^3^ Genome Institute of Singapore, Singapore, Singapore; ^4^ Cardiovascular Research Institute , National University Health System, Singapore, Singapore; ^5^ Centre for Quantitative Medicine, Duke-National University of Singapore (NUS) Medical School, National University of Singapore, Singapore, Singapore; ^6^ Department of Neurology, National Neuroscience Institute, Singapore, Singapore; ^7^ Ministry of Health Holdings, Singapore, Singapore; ^8^ Department of Diagnostic Radiology, Changi General Hospital, Singapore, Singapore; ^9^ Department of Surgery, Changi General Hospital, Singapore, Singapore; ^10^ Department of Urology, Changi General Hospital, Singapore, Singapore; ^11^ Department of Laboratory Medicine, Changi General Hospital, Singapore, Singapore; ^12^ Duke-National University of Singapore (NUS) Medical School, Singapore, Singapore; ^13^ Department of Cardiology, Changi General Hospital, Singapore, Singapore

**Keywords:** hyperaldosteronism, adrenalectomy, secondary hypertension, adrenal vein sampling (AVS), myocardial strain analysis, ejection fraction (EF)

## Abstract

**Introduction:**

Primary aldosteronism (PA) is associated with increased risk of cardiovascular events. However, treatment of PA has not been shown to improve left ventricular (LV) systolic function using the conventional assessment with LV ejection fraction (LVEF). We aim to use speckle-tracking echocardiography to assess for improvement in subclinical systolic function after treatment of PA.

**Methods:**

We prospectively recruited 57 patients with PA, who underwent 24-h ambulatory blood pressure (BP) measurements and echocardiography, including global longitudinal strain (GLS) assessment of left ventricle, at baseline and 12 months post-treatment.

**Results:**

At baseline, GLS was low in 14 of 50 (28.0%) patients. On multivariable analysis, GLS was associated with diastolic BP (*P =* 0.038) and glomerular filtration rate (*P =* 0.026). GLS improved post-surgery by −2.3, 95% CI: −3.9 to −0.6, *P =* 0.010, and post-medications by −1.3, 95% CI: −2.6 to 0.03, *P =* 0.089, whereas there were no changes in LVEF in either group. Improvement in GLS was independently correlated with baseline GLS (*P <* 0.001) and increase in plasma renin activity (*P =* 0.007). Patients with post-treatment plasma renin activity ≥1 ng/ml/h had improvements in GLS (*P =* 0.0019), whereas patients with persistently suppressed renin had no improvement. Post-adrenalectomy, there were also improvements in LV mass index (*P =* 0.012), left atrial volume index (*P =* 0.002), and mitral E/e’ (*P =* 0.006), whereas it was not statistically significant in patients treated with medications.

**Conclusion:**

Treatment of hyperaldosteronism is effective in improving subclinical LV systolic dysfunction. Elevation of renin levels after treatment, which reflects adequate reversal of sodium overload state, is associated with better systolic function after treatment.

**Clinical Trial Registration:**

www.ClinicalTrials.gov, identifier: NCT03174847.

## Introduction

Up to 20% of all patients with hypertension have primary aldosteronism (PA), making it the most common treatable cause of hypertension (1). Compared with patients with essential hypertension (EH) matched for age, gender, and blood pressure (BP), patients with PA are at higher risk of cardiovascular disease, renal failure, and poorer quality of life (2–4), attributed to the direct deleterious effects of aldosterone. Hyperaldosteronism induces left ventricular (LV) hypertrophy and fibrosis, which leads to LV remodeling and dysfunction. Patients with PA have increased LV mass index (LVMI), wall thickness, concentric remodeling, and LV diastolic dysfunction, which can be reversed with treatment (5). However, there are limited data on benefits of treatment on systolic function.

Systolic function is usually assessed with LV ejection fraction (LVEF), which has poor sensitivity, particularly in the presence of LV hypertrophy (6). LVEF impairment only occurs in end stage hypertensive heart disease. Other limitations include suboptimal reproducibility, inability to reflect regional LV function, and being a volume-centric measurement. Previous studies using LVEF have not found differences in patients with PA, compared with those with EH (7). In contrast, systolic function can also be assessed using myocardial strain, which represents a change in myocardial length from relaxed to contractile state, and assessed in different spatial components, e.g., longitudinal, circumferential, or radial. Myocardial strain is most commonly assessed using speckle-tracking echocardiography (8) on conventional B-mode echocardiography images, which is widely available. Speckle-tracking echocardiography offers high temporal and spatial resolutions, with good correlation with tagged magnetic resonance imaging, the reference method of strain assessment (9). Echocardiography-measured peak systolic global longitudinal strain (GLS) has been most extensively studied and can demonstrate impairment of systolic function before reduced LVEF.

Chen and colleagues demonstrated that patients with PA have impaired LV systolic function using GLS, compared with patients with EH (8), but did not perform repeat assessment after treatment. Although PA can be treated with mineralocorticoid (MR) antagonists, patients with unilateral PA can be offered curative adrenalectomy. Recent studies have found that surgery ameliorated the excess risk of cardiovascular events (10) and progression of renal disease (11). However, in patients with PA treated medically, the excess risk was only ameliorated in patients with unsuppressed renin levels post-treatment, suggesting that adequate medical treatment is important to reverse the deleterious effects of hyperaldosteronism. Hence, we conducted a prospective study in patients with PA, to assess for changes in LV GLS after both surgical and medical treatment.

## Methods

We prospectively recruited 57 patients with PA from February 2017 to October 2019 in Changi General Hospital, Singapore, in PA_PACES study (Primary Aldosteronism Prospective study Assessing Cardiovascular, Endothelial and other outcomes post-Surgical and medical treatment). The study was approved by local ethics committee, and informed consent was obtained from all patients (Clinicaltrials.gov:NCT03174847). Detailed baseline demographic characteristics and medical history were recorded.

### Diagnostic Tests for PA

Inclusion criteria were age 18 years and older and diagnosis of PA in accordance with the Endocrine Society guidelines (12). Exclusion criteria were patients with a terminal condition or glucocorticoid-remediable hyperaldosteronism. Plasma aldosterone concentration (PAC) and plasma renin activity (PRA) were measured as previously reported (13). Before hormonal measurements, MR antagonists were withdrawn at least 6 weeks in all patients, whereas medications that may interfere with PAC and PRA (e.g., ACE inhibitors and diuretics) were withdrawn at least 2 weeks when possible. Patients with aldosterone-renin ratio (ARR) greater than 554 (pmol/L per ng/ml/h) underwent confirmatory testing with seated intravenous saline infusion test, and all patients had a post-saline PAC of ≥138 pmol/L.

### Subtype Tests

Thin-sliced computed tomography (CT) of the adrenal glands was performed in all patients. In patients keen to undergo unilateral adrenalectomy, adrenal vein sampling (AVS) was performed sequentially under continuous corticotropin infusion by an experienced interventional radiologist (KSN) in majority of cases (14). Samples were taken from both adrenal veins and infrarenal vena cava (peripheral vein). AVS was successful if cortisol levels in both adrenal veins were at least three times that of peripheral vein. Lateralization ratio was determined by the higher adrenal aldosterone-cortisol ratio, divided by the contralateral adrenal aldosterone-cortisol ratio. Lateralization ratio of >4 was consistent with unilateral PA and of <3 was consistent with bilateral PA. Patients with ratio between 3 and 4 were discussed at a multidisciplinary meeting to determine subtype diagnosis and management. Patients treated surgically underwent unilateral adrenalectomy *via* minimally invasive transabdominal approach.

### Ambulatory Blood Pressure and 2D Echocardiography

All patients were scheduled to undergo a 24-h ambulatory BP monitoring and two-dimensional (2D) echocardiography (2DE) at baseline and 12 months post-treatment. Echocardiographic ultrasound systems equipped with speckle tracking, Philips, and General Electric were used to perform echocardiographic examinations. Transthoracic echocardiographic images were acquired at the enrolling centre following a study-specific acquisition protocol. Conventional measurements were analyzed by an independent core laboratory in our clinical measurement unit comprising senior sonographers. LV dimensions, septal, and posterior wall thickness were measured *via* the parasternal long axis view in accordance with the guidelines (15). Echocardiographic LVMI was calculated by applying the ASE M-mode equation. LVEF and left atrial volume index (LAVI) were measured by biplane area–length method (16). Speckle-tracking results were analyzed offline using a semi-automated algorithm (Cardiac Performance Analysis, a module of TomTec Arena; TomTec Imaging Systems, Unterschleissheim, Germany). The endocardial border was automatically detected and then tracked throughout the cardiac cycle using speckle-tracking technology. Manual adjustments were applied when needed to optimize boundary position only when necessary. GLS was calculated as the average of the magnitude of peak longitudinal strain from 17 ventricular segments, which were obtained from apical four-chamber, three-chamber, and two-chamber views (17). All strain analyses were performed by an experienced sonographer who was familiar with strain analysis and blinded to the clinical status of the patients. Normal LV GLS at our laboratory is less than −18%. Intra- and inter-observer reproducibility was estimated in 10 randomly selected subjects, and the coefficients of variation for GLS were 4.2% and 7.5% respectively.

### Outcomes

After at least 6 months post-treatment, all patients were reassessed for biochemical and clinical outcomes according to Primary Aldosteronism Surgery Outcome (PASO) consensus (18). Antihypertensive medications were recorded as number of medications and defined daily dosage (DDD) (https://www.whocc.no/atc_ddd_index/). Clinic BP was taken with an automated machine in all patients after five minutes of rest, from the arm in a seated position. In patients without a pre- or post-treatment 24-h ambulatory BP, clinic BP readings were used to calculate changes after treatment. Cure of hypertension was defined as post-treatment ambulatory daytime systolic BP below 135 mmHg and diastolic BP below 85 mmHg (systolic BP below 140 mmHg and diastolic BP below 90 mmHg, if clinic BP was used), without use of antihypertensive medications.

### Statistical Analysis

Statistical analysis was conducted using R (a language and environment for statistical computing; R Foundation for Statistical Computing, Vienna, Austria; https://www.R–project.org/). Continuous variables were expressed as a mean and standard deviation (SD) or median, minimum and maximum, or first and third quartile, as appropriate, and categorical variables were presented as number (percent). Groups were compared using independent samples *t*-test or Mann–Whitney U-test for continuous variables and chi-squared test or Fisher exact test for categorical variables, as appropriate. Post-treatment changes were compared using paired *t*-test or Wilcoxon signed-rank test for continuous variables and McNemar’s test for categorical variables, as appropriate. Univariate linear regression analysis was performed to investigate the association between GLS and clinical parameters at baseline. Significant determinants in the univariate linear regression analysis (*P <* 0.1) were then examined using multivariable linear regression analysis. This was similarly conducted to test for association between change in GLS and clinical parameters. Significance level was set at *P-*value of <0.05.

## Results

**Figure 1:**
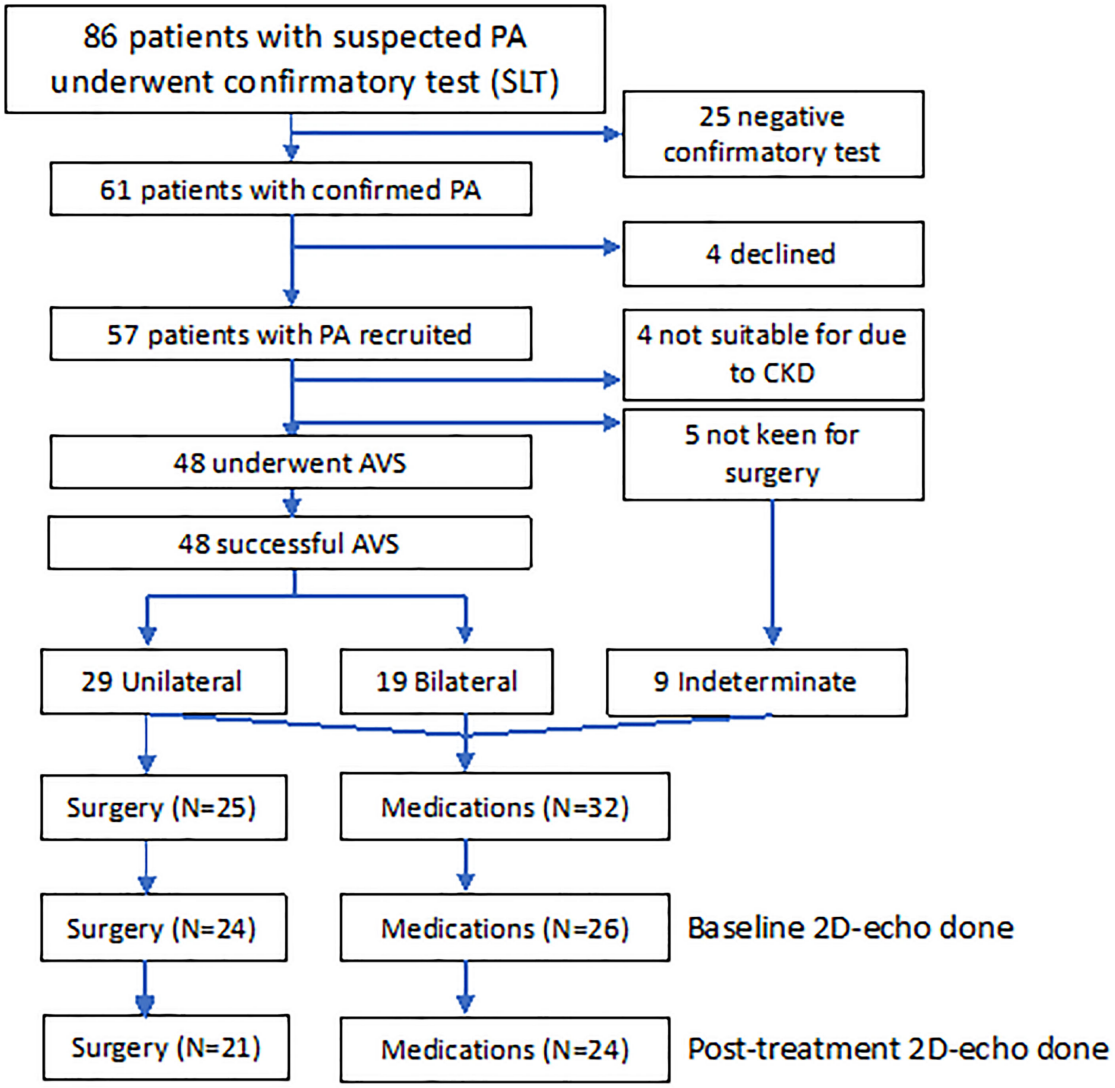
Consort diagram of 57 patients with PA recruited into study treated with adrenalectomy (n = 25) and medications (n = 32). AVS, adrenal vein sampling; CKD, chronic kidney disease; SLT, saline-loading test.

Eighty-six consecutive patients with ARR of >554 underwent confirmatory saline-infusion test, whereas two patients had PAC of >554 pmol/L, suppressed PRA, and spontaneous hypokalemia and did not require confirmatory testing. Of 61 patients confirmed with PA, we recruited 57 patients, mean age 54.8 ± 11.0 years, and 17 females (29.8%) (**Figure 1**). Four patients had chronic kidney disease, whereas five patients were not keen for adrenalectomy. The remaining 48 patients underwent AVS, with all patients having successful AVS. Among 29 patients with lateralization on AVS, 25 patients proceeded to unilateral adrenalectomy, whereas four patients opted for medical treatment despite lateralization on AVS: one for personal reasons and three had lateralization ratios of 4.0, 4.1, and 4.1, of which two patients’ AVS lateralized to the opposite side of an adrenal adenoma. Hence, 32 patients were treated with medications: 28 patients with spironolactone, and four patients with eplerenone and/or amiloride. The final median daily dose of spironolactone in patients medically treated was 37.5 (IQR: 25–75) mg. Patients treated with surgery were younger, had more severe hypokalemia, and were less likely to have ischemic heart disease and hyperlipidemia (**Table S1**) (19). In addition, patients treated surgically had a higher baseline daytime systolic and diastolic BP, 153 ± 16 mmHg and 94 ± 9 mmHg, respectively, compared with those on medications, 144 ± 12 and 85 ± 10 mmHg, respectively, *P =* 0.025 and *P =* 0.001.

### 2DE Parameters and Changes With Treatment

At baseline, 50 patients underwent 2DE (**Figure 1**). Baseline GLS was low (above −18) in 14 (28.0%) patients, of which 8 of 24 (33.3%) were treated surgically, and 6 of 26 (23.1%) were treated medically, with no statistical difference between the groups, *P =* 0.53. Absolute baseline GLS also did not differ between the surgical and medical groups, −19.3 ± 3.5 versus −20.0 ± 3.1, respectively, *P =* 0.31.

**Figure 2:**
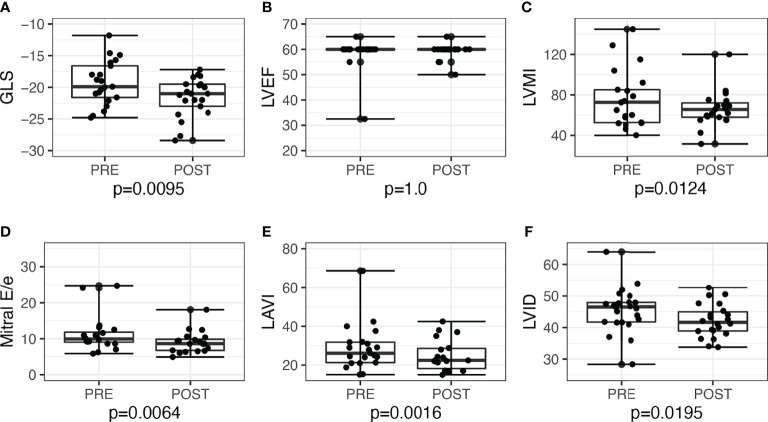
Echocardiographic parameters before and after adrenalectomy among patients with unilateral primary aldosteronism (n = 21). Improvement of left ventricular systolic function seen when assessed with **(A)** global longitudinal strain (GLS), but not with **(B)** left ventricular ejection fraction (LVEF). Improvements seen post-adrenalectomy in other parameters as well: **(C)** left ventricular mass index (LVMI); **(D)** mitral E/e’; **(E)** left atrial volume index (LAVI); **(F)** left ventricular internal dimension (LVID).

**Figure 3:**
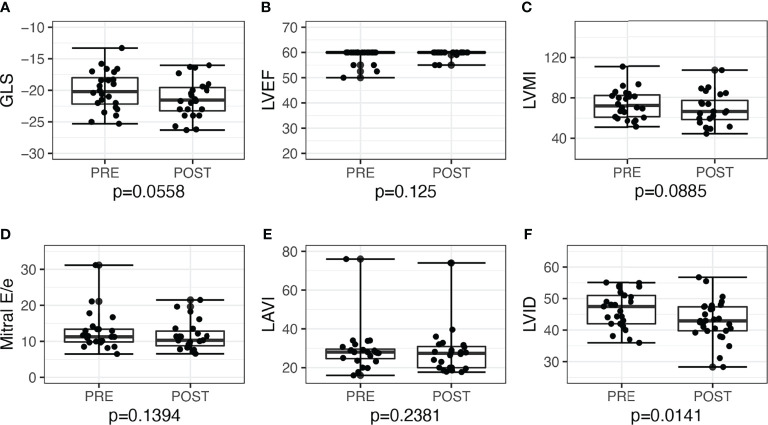
Echocardiographic parameters before and after medical treatment among patients with primary aldosteronism (n = 24). **(A)** Global longitudinal strain (GLS) but not in **(B)** left ventricular ejection fraction (LVEF). **(C)** Left ventricular mass index (LVMI); **(D)** mitral E/e’; **(E)** left atrial volume index (LAVI); **(F)** Left ventricular internal dimension (LVID).

LV GLS improved post-surgery by −2.3, 95% CI: −3.9 to −0.6, *P =* 0.010, and post-medications by −1.3, 95% CI: −2.6 to 0.03, *P =* 0.056. Overall, the proportion of patients with low GLS also improved post-treatment, from 12 (26.7%) to 5 (11.1%), *P =* 0.039. Post-adrenalectomy, there were improvements in LVMI by −10.0, 95% CI: −18.0 to −2.4, *P =* 0.012, LAVI by −5.0, 95% CI: −9.1 to −2.2, *P =* 0.002, and mitral E/e’ −1.9, 95% CI: −4.1 to −0.5, *P =* 0.006 (**Figure 2**). Post-medications, there was trend toward similar improvements in LVMI, LAVI, and mitral E/e’, but these did not reach statistical significance (**Figure 3**).

### Other Post-Treatment Changes

In both treatment groups, there were increases in potassium and PRA and declines in ARR and eGFR post-treatment (**Table 1**). In addition, PAC levels decreased in patients post-surgery. Surgical patients had higher baseline BP, and there was improvement in daytime and nighttime systolic and diastolic BP (all *P* ≤ 0.01) post-surgery. Medical patients did not have significant BP decline post-medications. However, post-treatment BP achieved was similar in both groups. Patients treated with surgery showed reduction in number of antihypertensive medications by 1.0 antihypertensive medication, 95% CI: 0.8 to 1.7, *P <* 0.001, wereas patients with medications had an increase by 1.0 antihypertensive medication, 95% CI: 0.02 to 0.9, *P =* 0.033.

**Table 1:** Changes in biochemical, 24-h ambulatory blood pressure and echocardiographic parameters from baseline to 12 months after surgical and medical treatment in patients with primary aldosteronism.

Variable	Surgical, n = 25					Medical, n = 32				
	n	Pre	Post	Diff (95% CI)	p-value	n	Pre	Post	Diff (95% CI)	p-value
Potassium, mmol/L	25	3.7 ± 0.4	4.3 ± 0.4	0.6 (0.4, 0.8)	<.0001	32	3.7 ± 0.4	4.1 ± 0.6	0.4 (0.1, 0.6)	0.005
eGFR	24	85.4 (51.5 to 111.1)	81.2 (31.6 to 106.5)	−5.18 (−10.6, −0.70)	0.037	32	76.2 (28.4 to 101.8)	67.1 (11.6 to 97)	−5.01 (−11, −2.51)	0.003
PAC	25	948 (388 to 1939)	552 (213 to 2354)	−804 (−1030, −672)	<.0001	24	491 (252 to 1496)	363 (139 to 2314)	−82 (−171, 397)	0.89
PRA	25	0.6 (0.2 to 2.3)	1.8 (0.6 to 19)	1 (0.8, 4.7)	<.0001	30	0.6 (0.2 to 2.5)	1.3 (0.6 to 14)	0.9 (0.9, 3.1)	<.0001
ARR	25	1790 (554 to 8367)	79.8 (7 to 205)	−1,689 (−3,295, −1,600)	<.0001	24	1229 (216 to 7133)	448 (8 to 2409)	−565 (−1861, −522)	<.0001
Daytime Systolic BP, mmHg	22	151.8 ± 16.4	136.4 ± 14.1	−15.4 (−25.0, −5.9)	0.003	26	142.1 ± 12.1	137.2 ± 17.1	−4.9 (−12, 2.2)	0.17
Daytime Diastolic BP, mmHg	22	93.1 ± 9.3	86.5 ± 11.1	−6.7 (−11.6,−1.8)	0.010	26	85.4 ± 10.5	85.2 ± 11	−0.2 (−4, 3.6)	0.92
Night–time Systolic BP, mmHg	22	145.1 ± 14.6	130.3 ± 14.9	−14.8 (−22.0, −7.6)	<.0001	26	135.8 ± 16.0	131.3 ± 27.2	−4.5 (−13.5, 4.5)	0.31
Night–time Diastolic BP, mmHg	22	87.4 ± 8.3	78 ± 10.3	−9.4 (−14.3, −4.5)	<.0001	26	79.3 ± 1 0.5	77.8 ± 13.4	−1.5 (−6.2, 3.2)	0.53
LVEF, %	21	60 (32.5 to 65)	60 (50 to 65)	0 (−1.5, 2.7)	1.0	24	60 (50 to 60)	60 (55 to 60)	0 (0.0, 1.4)	0.13
IVS, mm	21	9.22 ± 2.1	9.25 ± 2.24	0.03 (−0.53, 0.58)	0.92	24	9.22 ± 1.59	8.94 ± 1.62	−0.28 (−1.03, 0.46)	0.44
LVPW, mm	21	8.96 ± 1.94	8.51 ± 1.93	−0.45 (−1.07, 0.16)	0.14	24	8.27 ± 1.17	8.42 ± 1.3	0.15 (−0.46, 0.76)	0.62
LVMI, g/m^2^	21	72.6 (40 to 144.9)	65.5 (31.4 to 120)	−10.0 (−18.0, −2.4)	0.012	24	72 (51 to 111)	66 (44 to 107)	−5 (−11.2, 0.3)	0.089
RWT	21	0.40 ± 0.09	0.42 ± 0.14	0.02 (−0.03, 0.06)	0.45	24	0.37 ± 0.08	0.40 ± 0.09	0.03 (−0.02, 0.07)	0.21
GLS	21	−19.3 ± 3.5	−21.6 ± 3.0	−2.3 (−3.9, −0.6)	0.010	24	−20.0 ± 3.1	−21.3 ± 3.0	−1.3 (−2.6, 0.03)	0.056
LAVI, ml/m^2^	21	26.1 (15.1 to 68.6)	22.1 (11.5 to 42.5)	−5 (−9.1, −2.2)	0.002	24	28.0 (16.0 to 76.0)	27.4 (17.7 to 74.0)	−0.7 (−3.4, 1.4)	0.24
LVID, mm	21	45.2 ± 7.2	42.3 ± 5.3	−2.9 (−5.2, −0.5)	0.020	23	46.2 ± 5.9	43 ± 6.9	−3.3 (−5.8, −0.7)	0.014
Mitral E/e’	20	10 (5.9 to 24.7)	8.6 (4.9 to 18.1)	−1.9 (−4.1, −0.5)	0.006	23	11.3 (6.5 to 31.2)	10.3 (6.6 to 21.5)	−1.5 (−2.8, 0.5)	0.14
Patients with impaired LVEF <50%, %	21	2 (9.5%)	1 (4.8%)	–	0.32	24	0 (0%)	0 (0%)	–	NA
Patients with low GLS >–18, %	21	6 (28.6%)	1 (4.8%)	–	0.025	24	6 (25.0%)	4 (16.7%)	–	0.32
Antihypertensive medications, number	25	2 (1 to 5)	1 (0 to 2)	−1 (−1.7, −0.8)	<.0001	32	2 (0 to 4)	3 (0 to 5)	1 (0.02, 0.9)	0.033
Antihypertensive medications, number, DDD	25	3 (0.4 to 10.8)	1 (0 to 6)	−2 (−3.2, −1.3)	<.0001	32	2 (0 to 4)	3 (0 to 5)	0.2 (−0.7, 0.6)	0.80

ARR, aldosterone-renin ratio; BP, blood pressure; DDD, defined daily dose; E/e’, early diastolic transmitral and myocardial velocity on tissue Doppler imaging ratio; eGFR, estimated glomerular filtration rate; IVS, interventricular septum; LAVI, left atrium volume index; GLS, global longitudinal strain; LVEF, left ventricular ejection fraction; LVID, left ventricular internal dimension; LVMI, left ventricular mass index; LVPW, left ventricular posterior wall thickness; PAC, plasma aldosterone concentration; PRA, plasma renin activity; RWT, relative wall thickness in diastole.

Continuous variables reported as mean ± standard deviation or median (minimum to maximum) and compared between pre and post-treatment using paired t-test or Wilcoxon signed-rank test depending on normality assumption; categorical variable presented as frequency (%), and compared between pre and post treatment using McNemar’s test. NA, Not Applicable.

### Linear Regression Analysis

On univariate analysis, baseline GLS was associated with baseline diastolic BP (**Table 2**), with an unadjusted coefficient 0.090, 95% CI: 0.005 to 0.175, *P =* 0.038. On multivariable analysis, after including baseline diastolic BP, both baseline diastolic BP, adjusted coefficient 0.090, 95% CI: 0.005 to 0.175, *P =* 0.038, and baseline glomerular filtration rate (GFR), adjusted coefficient −0.059, 95% CI:–0.111 to −0.008, *P =* 0.026, were correlated with baseline GLS.

**Table 2:** Univariate and multivariable linear model of baseline global longitudinal strain (GLS) in all patients treated for primary aldosteronism (n = 45).

Variable	Univariate		Multivariable *	
	Un-Adjusted BetaCoefficient (95% CI)	*p*-value	Adjusted BetaCoefficient (95% CI)	*p*-value
Female gender	−0.200 (−2.44, 2.042)	0.86	−0.114 (−2.282, 2.054)	0.92
Age	−0.004 (−0.096, 0.089)	0.94	0.065 (−0.039, 0.169)	0.22
BMI, g/m2	0.056 (−0.144, 0.256)	0.58	0.009 (−0.190, 0.208)	0.93
Systolic BP, mmHg	0.013 (−0.051, 0.078)	0.68	−0.049 (−0.128, 0.031)	0.23
**Diastolic BP, mmHg**	**0.090 (0.005, 0.175)**	**0.038**	**0.090 (0.005, 0.175)**	**0.038**
Baseline antihypertensive medications, number	0.032 (−0.852, 0.916)	0.94	0.376 (−0.523, 1.276)	0.40
Baseline potassium	−1.45 (−3.90, 0.998)	0.24	−1.26 (−3.64, 1.121)	0.29
**Baseline eGFR**	−0.034 (−0.087, 0.018)	0.19	**−0.059 (−0.111, −0.008)**	**0.026**
Baseline log PAC	−0.177 (−2.07, 1.719)	0.85	−0.734 (−2.62, 1.154)	0.44
Baseline log PRA	−1.150 (−5.749, 3.45)	0.62	−0.813 (−5.274, 3.649)	0.72
Baseline log ARR	0.139 (−1.07, 1.343)	0.82	−0.156 (−1.35, 1.041)	0.79

ARR, aldosterone-renin ratio; BP, blood pressure; eGFR, estimated glomerular filtration rate; PAC, plasma aldosterone concentration; PRA, plasma renin activity.

Linear regression analysis was performed to calculate the beta coefficients and 95% confidence intervals.

* Adjusted for DBP. Bold values are statistically significant.

On univariate analysis, improvement in GLS was associated with baseline daytime diastolic BP, baseline GLS, and change in log PRA (**Table 3**). On multivariable analysis, only baseline GLS, adjusted coefficient −0.534, 95% CI: −0.781 to −0.282, *P <* 0.001, and increase in log PRA, adjusted coefficient −1.428, 95% CI:–2.438 to −0.417), *P =* 0.007, were associated with improvement in GLS.

**Table 3:** Univariate and multivariable linear model of Δglobal longitudinal strain (GLS) before and after treatment in all patients treated for primary aldosteronism (n = 45).

Variable	Univariate		Multivariable*	
	Un-Adjusted BetaCoefficient (95% CI)	p-value	Adjusted BetaCoefficient (95% CI)	p-value
Female Gender	0.593 (−1.66, 2.843)	0.60		
Age, years	0.032 (−0.059, 0.124)	0.48		
BMI, kg/m^2^	−0.067 (−0.271, 0.137)	0.51		
Baseline systolic BP, mmHg	−0.044 (−0.109, 0.022)	0.19		
Baseline diastolic BP, mmHg	−0.089 (−0.179, 0.001)	0.053	−0.068 (−0.143,.008)	0.077
Baseline antihypertensive medications, number	−0.216 (−1.12, 0.692)	0.63		
Baseline potassium	0.490 (−2.05,3.028)	0.70		
Baseline eGFR	−0.011(−0.067,0.043)	0.68		
Baseline log PAC	−0.118 (−2.08, 1.845)	0.90		
Baseline log PRA	1.325 (−3.41, 6.06)	0.56		
Baseline log ARR	−0.235 (−1.50,1.026)	0.71		
**Baseline GLS**	**−0.610 (−0.864, −0.355)**	**<.0001**	**−0.534 (−0.781, −0.282)**	**<.0001**
Δ potassium	0.607 (−0.925, 2.138)	0.43		
Δ eGFR	0.023 (−0.069,0.114)	0.62		
Δ systolic BP, mmHg	0.007 (−0.044,0.058)	0.77		
Δ diastolic BP, mmHg	0.080 (−0.014, 0.174)	0.094		
Δ log PAC	0.120 (−1.340, 1.580)	0.87		
**Δ log PRA**	**−1.36 (−2.62, −0.097)**	**0.036**	**−1.428 (−2.438, −0.417)**	**0.007**
Δ LVMI	0.045 (−0.021, 0.110)	0.18		
Δ LAVI	−0.047 (−0.195, 0.100)	0.52		
Δ antihypertensive medications, number	0.159 (−0.562, 0.879)	0.66		

ARR, aldosterone-renin ratio; BP, Blood Pressure; eGFR, estimated glomerular filtration rate; PAC, plasma aldosterone concentration; PRA, plasma renin activity.

Linear regression analysis was performed to calculate the beta coefficients and 95% confidence intervals.

* Backward selection criteria: significant level of stay = 0.1.

### Changes of GLS in Subgroups

**Figure 4:**
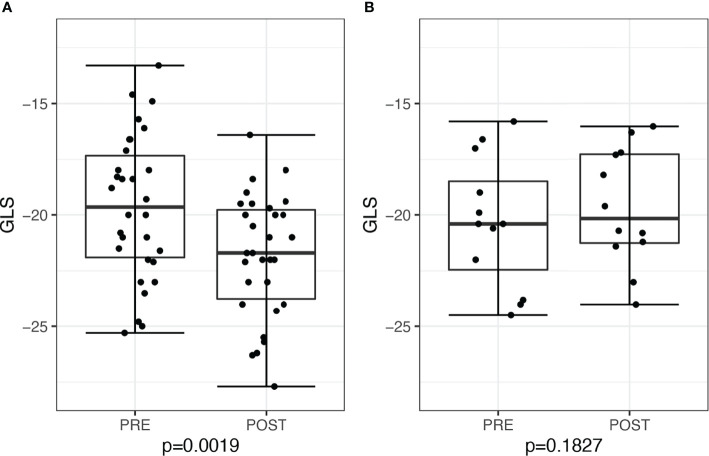
Global longitudinal score (GLS) in patients before and after treatment for primary aldosteronism stratified by post-treatment plasma renin activity (PRA). **(A)** Patients with post-treatment PRA >1 ng/ml/h (n = 30) and **(B)** patients with post-treatment PRA ≤1 ng/ml/h (n = 13).

When stratified by post-treatment renin response, there was a significant decline in post-treatment GLS of −2.45, 95% CI: −3.48 to −0.84, *P =* 0.0019, among 30 patients who achieved post-treatment PRA ≥1 ng/ml/h, whereas there was no significant change in GLS of −0.7, 95% CI −2.53 to 0.72, *P* = 0.18, among 13 patients with persistent PRA <1 ng/ml/h (six treated with surgery and seven treated with medications) (**Figure 4**). We further restricted analysis only to medically–treated patients with similar results, demonstrating in 15 patients with PRA ≥1ng/ml/h an improvement in GLS of −1.7, 95% CI: −3.4 to −0.1, *P =* 0.042, compared with no change in seven patients with persistent PRA <1 ng/ml/h, of +0.1, 95% CI: −2.8 to 3.1, *P* = 0.91.

When stratified by post-treatment BP changes, patients who showed improvements in both systolic and diastolic BP had improvements in GLS of −2.1, 95% CI: −3.6 to −0.7, *P =* 0.006, whereas patients without improvement in BP had a tendency toward improvement in GLS of −1.1, −2.5 to 0.2, *P =* 0.088.

### PASO Outcome

Among 25 patients who underwent surgery, all 25 patients had complete biochemical cure as defined by PASO, with resolution of hypokalemia and normalization of ARR. Three patients had complete clinical success, 17 had partial clinical success, and five had no clinical response. Among 32 patients treated medically, 12 had persistent renin suppression (PRA <1ng/ml/h), whereas four had hypokalemia at their 1-year clinic visit.

## Discussion

We demonstrated that treatment of PA can improve subclinical LV systolic function, and this improvement was associated with a rise in renin levels. This provides further support that reversal of renin suppression is important in ameliorating the excess cardiovascular risk seen in PA. In addition, baseline GLS was associated with baseline renal function and diastolic BP, suggesting either an interplay between cardiac and renal dysfunction (cardiorenal syndrome) or more likely hyperaldosteronism contributing to pathology in both organs. Assessment with speckle-tracking echocardiography was more sensitive in detecting improvement in systolic function compared with conventional LVEF assessment. In our study, surgery led to greater improvements in cardiac function compared with medical treatment, which underlines the importance of subtyping PA and offers curative adrenalectomy for unilateral disease.

Although patients with PA are at increased risk of cardiac failure, there are limited data to show that treatment of PA can improve LV systolic function (20). A previous study found that patients with PA had a subclinical LV systolic dysfunction compared with those with EH, using GLS (8). We have now demonstrated improvement of LV systolic function with adrenalectomy for PA and, to a lesser extent, with medications. Similar to previous studies, we did not find changes in LVEF post-treatment (7, 13), highlighting the limited sensitivity of LVEF. GLS analysis using 2D speckle-tracking echocardiography is more sensitive and more reproducible (9), leading expert committees to recommend its routine use to monitor patients during chemotherapy for cardiotoxicity (21). Improvements in GLS have also been observed in treatment of patients with other forms of secondary hypertension. Dobrowolski and colleagues demonstrated that resection of a pheochromocytoma or paraganglioma led to improvements in LV subclinical systolic dysfunction assessed by GLS, highlighting the deleterious effects of catecholamine excess on the myocardium (22).

Of particular interest, we found that improvements in systolic function correlated with reversal of renin suppression and baseline GLS. Studies by Hundemer and colleagues found that patients with PA treated with medications with persistent renin suppression (PRA < 1 ng/ml/h) remained at high cardiovascular risk of ischemic events, atrial fibrillation, and cardiac death, whereas those with unsuppressed renin (PRA ≥ 1 ng/ml/h) had similar risk to patients with EH (10, 23). In our study, improvement in GLS was only observed in the group of patients with post-treatment PRA ≥ 1 ng/ml/h, and not among those with persistent renin suppression. These findings support the notion that adequate reversal sodium and volume overload, reflected by a rise in PRA, is important to improve LV systolic function and lower the risk of further cardiac sequelae. In addition, when stratified by BP response, we found that patients with improvement in BP had improvement in subclinical systolic function. Our patients treated with surgery had higher baseline BP and greater BP reductions post-treatment, although the final BP achieved was similar in both groups. This may explain the greater improvement seen in the surgery group. These findings suggest that, in patients with EH, improvement of BP control may have similar positive effects on cardiac function, whereas in patients with PA, it highlights the importance of attaining both BP and biochemical control.

We also found that, at baseline, lower GLS was associated with lower GFR and higher diastolic BP. High diastolic BP likely leads to increased afterload, resulting in poorer systolic function (24). The association of GLS with GFR is particularly interesting. Cardiorenal syndrome is a classification of patients with both organ dysfunction based on the presumed primary diseased organ (25). In PA, dysfunction in both organs is likely due to a common pathway of aldosterone excess. Elevated aldosterone levels lead to myocyte hypertrophy, chronic inflammation, and dysregulation of the extracellular matrix (20). Resultant LV hypertrophy and remodeling predisposes to systolic and diastolic dysfunction, and patients with PA are twice as likely to develop cardiac failure (3), whereas treatment reduces the incidence of heart failure (13). Similarly, hyperaldosteronism leads to glomerular hyperfiltration, falsely elevated GFR (4), increased risk of fibrosis, and renal disease. Most of our patients had normal GFR at baseline, but significant decline in GFR post-treatment was observed, due to unmasking of the underlying renal impairment. Our findings highlight a current challenge in managing these patients, with subclinical multiorgan dysfunction early in the disease. GLS has been shown to identify subclinical LV dysfunction, before onset of heart failure with reduced EF (9), and has important prognostic value (26). Hence, our study suggests that GLS may be useful in detecting more patients with subclinical LV dysfunction compared with the conventional LVEF.

In addition to improvement of GLS, other cardiac benefits were seen in our patients post-adrenalectomy. Regression of LVH is concordant with findings from a recent meta-analysis (5). Reduction in LA volume index could be due to improvement in LV diastolic function (reflected by decline in mitral E/e’) and decreased LVMI that improves ventricular compliance. Aldosterone has been shown to induce atrial fibrillation through electrophysiological dysfunction, inflammation, and vascular remodeling (27), and treatment of PA can reduce risk of new onset atrial fibrillation (3, 23). Overall, we found that surgery led to greater improvement in cardiac parameters compared with medical treatment, consistent with some, but not all, previous studies (28, 29). In addition to differences in extent of BP improvement as mentioned earlier, there are other possible reasons for this. First, some studies have shown that a longer duration of follow-up eventually lead to similar outcomes, suggesting that the benefits of medical treatment may be less immediate (5). In a recent study, Chen and colleagues found that GLS improved after adrenalectomy but not after medical treatment for PA, whereas we found a trend toward improvement in GLS in patients after medical treatment. This may be because our study had a longer follow-up duration of 12 months compared with 3–6 months in the earlier study. Second, our medically treated patients had improvements in serum potassium and PRA, but no changes to BP. More aggressive up-titration of MR antagonists has been shown to provide greater improvements in BP and albuminuria (30) and may have led to improvements in subclinical systolic dysfunction in our patients. It has to be noted that medical treatment may be fraught with dose-limiting side effects, leading to suboptimal dosages and efficacy, as other investigators (31) and we have previously reported (32). Finally, current MR antagonists may not completely block aldosterone effects or concomitant glucocorticoid excess, which can occur in PA (13).

Our prospective cohort study of patients with confirmed PA had several strengths, including successful AVS in all patients, which allowed appropriate treatment according to underlying PA subtype. This high AVS success rates have been achieved only in a few centers worldwide (33) and were attained by an experienced and dedicated interventional radiologist (KSN) leading the team. We also performed pre- and post-treatment 24-h ambulatory BP measurements, which are superior to office BP for reflecting hemodynamic status. We recognize several limitations. First, we did not have information on dietary salt intake although this is likely high (34). Excessive dietary salt is important in cardiac deterioration induced by hyperaldosteronism (35), through sodium retention and volume expansion. However, we demonstrated that changes in renin levels, which reflect volume status, correlated with improvement in LV systolic dysfunction. Second, with a larger patient cohort, we may have been able to identify other variables correlated with GLS, such as severity of hyperaldosteronism. Using 24-h urine aldosterone may also be better to quantify the total aldosterone production (1). Third, we did not routinely assess for autonomous cortisol secretion. Using metabolome analysis, cortisol excess has been reported in PA and has been associated with increased LV hypertrophy (13, 36). Fourth, we did not have a control group of patients with EH for comparison, but a previous study has already demonstrated that patients with PA have an impaired LV systolic function compared with patients with EH with similar BP (8). Finally, although we found improved LV systolic dysfunction, further studies will be required to demonstrate that this improvement in LV GLS leads to clinical significant outcomes for patients with PA. However, there is currently evidence that LV GLS has important prognostic implications in other patient populations (21, 26).

In conclusion, we demonstrated that appropriate treatment of PA can improve LV subclinical systolic function, with either unilateral adrenalectomy or medical treatment after adequate MR blockade, as reflected by unsuppression of renin. Furthermore, the co-occurrence of cardiac and renal dysfunction highlights the various sequalae of hyperaldosteronism. This underlines the importance of early diagnosis and treatment of PA, to prevent further organ damage.

## Perspectives

**Figure 5:**
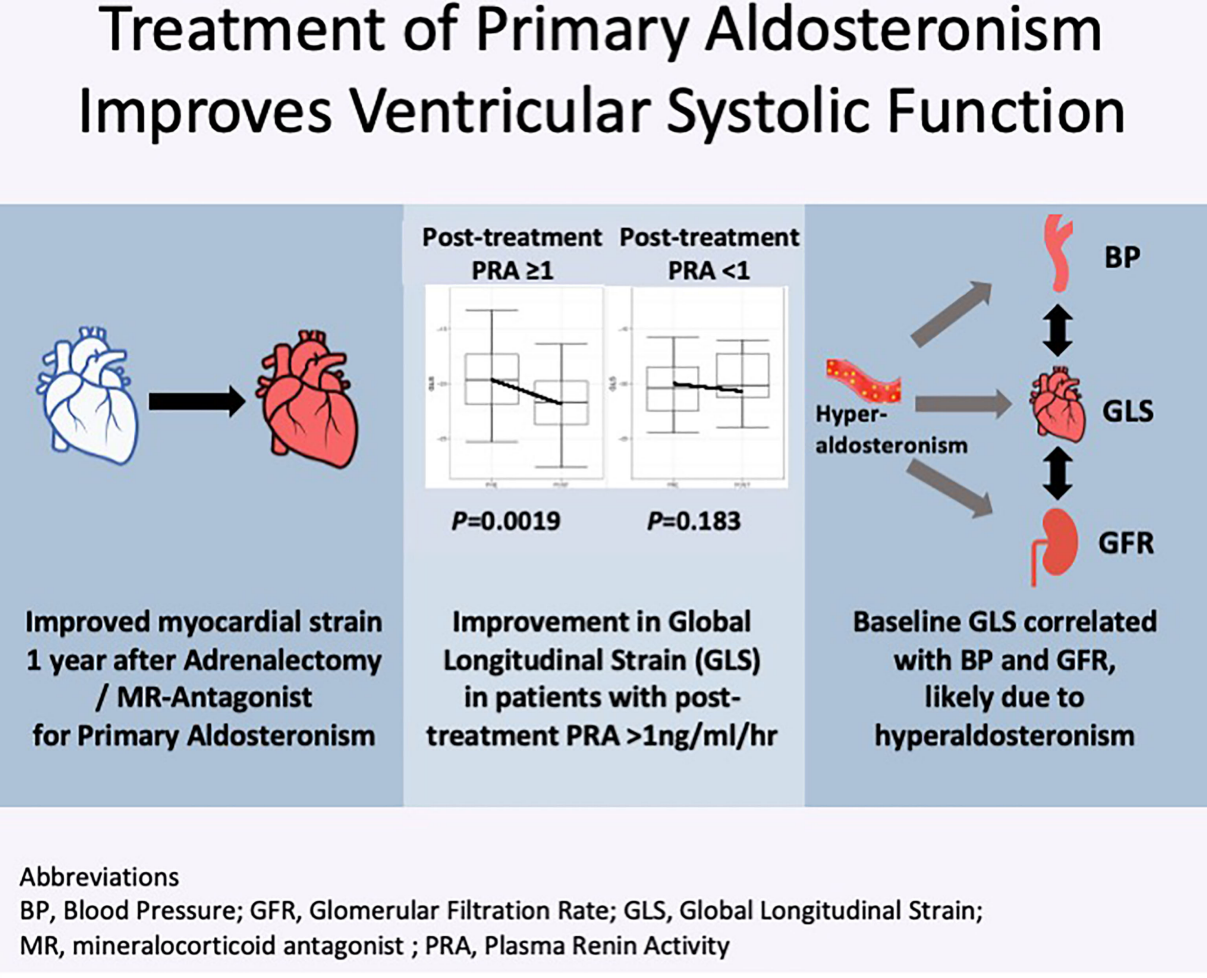
Visual Abstract.

We have shown with speckle-tracking echocardiography that patients with PA have subclinical LV systolic dysfunction (**Figure 5**). This is associated with impaired renal function, highlighting the myriad of complications attributed to hyperaldosteronism. Specific treatment of PA, with adequate reversal of renin suppression and sodium overload state, is able to improve subclinical LV systolic function. With PA affecting up to 20% of all patients with hypertension, this reinforces the call for increased screening and early treatment of primary aldosteronism.

## Data Availability Statement

The raw data supporting the conclusions of this article will be made available by the authors, without undue reservation.

## Ethics Statement

The studies involving human participants were reviewed and approved by SingHealth Institutional Review Board. The patients/participants provided their written informed consent to participate in this study.

## Author Contributions

TP, CC, and SCC had full access to the data in the study and take responsibility of the integrity of the data and accuracy of data analysis. TP, MZ, JK, LG, TK, WL, SS, VA, TT, ET, LM, and JY recruited patients in the study and performed data collection. TP, RF, YT, KT, SL, and S-CC worked on the study concept and design. TP, SS, TT, MC, KT, SL, and SCC were involved in data collection and analysis. TP, CC, TT, MC, and SCC were involved in drafting of the manuscript. All authors contributed to the article and approved the submitted version.

## Funding

This research was supported by grants from Changi Health Fund (CHF2016.02-P and CHF2018.03-S).

## Conflict of Interest

The authors declare that the research was conducted in the absence of any commercial or financial relationships that could be construed as a potential conflict of interest.

## Publisher’s Note

All claims expressed in this article are solely those of the authors and do not necessarily represent those of their affiliated organizations, or those of the publisher, the editors and the reviewers. Any product that may be evaluated in this article, or claim that may be made by its manufacturer, is not guaranteed or endorsed by the publisher.
