# Supplementary material for: Treatment of Primary Aldosteronism and Reversal of Renin Suppression Improves Left Ventricular Systolic Function
Source: Front Endocrinol (Lausanne). 2022 Jun 30;13:916744. doi: 10.3389/fendo.2022.916744 (PMC9279860; doi:10.3389/fendo.2022.916744)
Supplement: Supplementary file 1 [file DataSheet_1.pdf]

Supplementary Material

Table S1: Baseline characteristics of 57 patients with primary aldosteronism

	Total (N = 57)	Surgical (N = 25)	Medical (N = 32)	*P value*
Age (years)	54.8 ±11.0	49.9 ±11.5	58.6 ±9.0	0.006
Females (%) Gender	17 (29.8%)	9 (36.0%)	8 (25.0%)	0.40
BMI (kg/m^2)	26.9 ±4.8	27.3 ±5.1	26.6 ±4.6	0.60
Day–time Systolic BP, mmHg	147.4 ±14.8	152.5 ±15.9	143.1 ±12.4	0.021
Day–time Diastolic BP, mmHg	89.2 ±10.4	93.5 ±9	85.6 ±10.3	0.005
Night–time Systolic BP, mmHg	140.9 ±15.7	146 ±14.3	136.5 ±15.8	0.029
Night–time Diastolic BP, mmHg	83.1 ±10	87.3 ±8.1	79.5 ±10.2	0.004
Antihypertensive medications, number	2 (0 to 5)	2 (1 to 5)	2 (0 to 4)	0.71
Antihypertensive medications, DDD	2.8 (1.5 to 4.6)	3.0 (2.0 to 4.6)	2.4 (1.2 to 4.7)	0.43
Serum potassium, mmol/L	3.7 ±0.4	3.7 ±0.4	3.8 ±0.4	0.49
PAC, pmol/L	785 ±400	963 ±401	633 ±336	0.002
PRA, ng/ml/hr	0.6 ±0.4	0.5 ±0.3	0.6 ±0.5	0.28
ARR	2039 ±1832	2616 ±2074	1544 ±1457	0.034
Post saline–loading PAC, pmol/L	621 ±513	752 ±526	518 ±478	0.11
eGFR	78 ±18	82 ±16	75 ±19	0.14
Years of hypertension (N = 43)	12.2 ±8.4	10.7 ±7.0	13.7 ±9.6	0.26
Ischemic Heart Disease (%)	7 (12.3%)	1 (4.0%)	6 (18.8%)	0.092

Chronic Kidney Disease (%)	5 (8.8%)	1 (4.0%)	4 (12.5%)	0.26
Cerebrovascular Accident (%)	4 (7.0%)	3 (12.0%)	1 (3.1%)	0.19
Hyperlipidaemia (%)	28 (49.1%)	9 (36.0%)	19 (59.4%)	0.080
Diabetes Mellitus (%)	20 (35.1%)	9 (36.0%)	11 (34.4%)	0.90
Atrial Fibrillation (%)	3 (5.3%)	1 (4.0%)	2 (6.3%)	0.70
CT scan results (%)				
Unilateral adenoma	37 (64.9%)	20 (80.0%)	17 (53.1%)	0.14
Bilateral adenoma	2 (3.5%)	1 (4.0%)	1 (3.1%)	
Bilateral normal	16 (28.1%)	4 (16.0%)	12 (37.5%)	

ARR, aldosterone–renin ratio; BMI, Body Mass Index; BP, Blood Pressure; CT, Computed Tomography; DDD, Defined Daily Dose; eGFR, Estimated Glomerular Filtration Rate; PAC, plasma aldosterone concentration; PRA, plasma renin activity

Data given as mean ±standard deviation, median (minimum to maximum) or frequency (percent) as appropriate
